# 1,25-Dihydroxyvitamin D_3_ Induces LL-37 and HBD-2 Production in Keratinocytes from Diabetic Foot Ulcers Promoting Wound Healing: An In Vitro Model

**DOI:** 10.1371/journal.pone.0111355

**Published:** 2014-10-22

**Authors:** Irma Gonzalez-Curiel, Valentin Trujillo, Alejandra Montoya-Rosales, Kublai Rincon, Bruno Rivas-Calderon, Jeny deHaro-Acosta, Paulina Marin-Luevano, Daniel Lozano-Lopez, Jose A. Enciso-Moreno, Bruno Rivas-Santiago

**Affiliations:** 1 Medical Research Unit Zacatecas, Mexican Institute of Social Security-IMSS, Zacatecas, Mexico; 2 Department of Immunology, Faculty of Medicine, Autonomous University of San Luis Potosi, San Luis Potosi, Mexico; University Hospital Schleswig-Holstein, Campus Kiel, Germany

## Abstract

Diabetic foot ulcers (DFU) are one of the most common diabetes-related cause of hospitalization and often lead to severe infections and poor healing. It has been recently reported that patients with DFU have lower levels of antimicrobial peptides (AMPs) at the lesion area, which contributes with the impairment of wound healing. The aim of this study was to determine whether 1,25-dihydroxyvitamin D_3_ (1,25 (OH)_2_ D_3_) and L-isoleucine induced HBD-2 and LL-37 in primary cultures from DFU. We developed primary cell cultures from skin biopsies from 15 patients with DFU and 15 from healthy donors. Cultures were treated with 1,25 (OH)_2_D_3_ or L-isoleucine for 18 h. Keratinocytes phenotype was identified by western blot and flow cytometry. Real time qPCR for DEFB4, CAMP and VDR gene expression was performed as well as an ELISA to measure HBD-2 and LL-37 in supernatant. Antimicrobial activity, in vitro, wound healing and proliferation assays were performed with conditioned supernatant. The results show that primary culture from DFU treated with 1,25(OH)_2_D_3_, increased DEFB4 and CAMP gene expression and increased the production of HBD-2 and LL-37 in the culture supernatant. These supernatants had antimicrobial activity over *E. coli* and induced remarkable keratinocyte migration. In conclusion the 1,25(OH)_2_D_3_ restored the production of AMPs in primary cell from DFU which were capable to improve the in vitro wound healing assays, suggesting their potential therapeutic use on the treatment of DFU.

## Introduction

It is estimated that more than 356 million people worldwide have type 2 *diabetes mellitus* (NIDDM) and 15–25% of these patients will develop diabetic foot ulcerations (DFU) during their lifetime [1]. Peripheral neuropathy, vascular insufficiency and diminished immune response are major factors in the development of skin ulceration and infection in these patients [2]. Indeed, is estimated that 50% of these chronic ulcerations will become infected by several microorganism*s*, which together with vascular insufficiency and poor wound healing lead to high hospitalization rates and increased the probability of lower extremity amputation [3]. Successful treatment for DFU in developing countries relays essentially on the debridement of all necrotic tissue and infection control with systemic antibiotics [4], which are costly and not often effective. Therefore, new approaches for the creation of new drugs or adjuvants to improve the current therapy are urgently needed. The use of new molecules that have antimicrobial, angiogenic and wound healing activity are highly desirable for the DFU treatment.

Antimicrobial peptides (AMPs) are versatile molecules that have been strongly involved in the innate immunity through direct killing of invader microorganisms [5]. However this is not the only feature regarding these peptides; it has been widely described their ability to module the immune response through a variety of mechanisms, such as neutralization of endotoxins, chemotaxis, immature dendritic cell activation, cytokine induction [6], wound healing and pro-angiogenic properties [7] through a wide variety of receptors.

Although it has been reported the presence of many AMPs on healthy skin surface, two members can be highlighted: β-defensin and cathelicidin [8]. Originally identified because of their direct antimicrobial function, however, there is growing evidence that both peptides play an important additional role during the wound healing process [9], supporting processes such as proliferation, migration and differentiation of the dermal fibroblasts and epidermal keratinocytes by human β-defensin (HBD) -2 [10] or angiogenesis and keratinocyte migration by the cathelicidin (LL-37) [11], [12]. Studies performed in animal models have shown that higher expression of their homologous genes such as CRAMP and mBD-3 by the skin keratinocytes is related to bacterial infection resistance and a proper wound healing [13], [14].

We recently showed that patients with NIDDM have lower levels of CAMP (LL-37) and DEFB4 (HBD-2) gene expression in peripheral blood cells [15] which probably is translated into susceptibility to infectious diseases. Furthermore, it has been reported that the expression of DEFB4 is lower in DFU in comparison with healthy skin, interestingly most of the DFU patients showed the lack of CAMP gene expression [16], suggesting that low levels of this peptide contribute to poor wound healing in DFU patients.

AMPs could be induced through several Pathogen-Associated Molecular Patterns (PAMPs) such as lipoarabinomannan, lipopolysaccharides [17] and proinflammatory cytokines [18]. However, none of these molecules can be used as therapeutic inductor because they may induce undesirable side effects. Interestingly, it has been reported that L-isoleucine and several of its analogs can specifically induce β-defensin expression efficiently in epithelial cells [19], [20].

In the last decade the participation of the vitamin D_3_ in the epithelial immunity has been highlighted. Recent studies have shown that the treatment with vitamin D resulted in an increased LL-37 gene expression through the vitamin D receptor of cultured sebocytes [21]. Likewise, 1,25 (OH)_2_ D_3_ induces changes in the expression of LL-37 and HBD-2 in monocytes, macrophages, neutrophils and keratinocytes [22], [23].

Thus, we sought to determine whether L-isoleucine or 1,25 (OH)_2_ D_3_ may induced LL-37 and/or HBD-2 in primary cultures obtained from DFU and if these induced-peptides had antimicrobial and wound healing activities. The major finding of the present study was that 1,25(OH)_2_D_3_ restored the production of AMPs in primary cell from DFU contributing to improve the in vitro healing assays.

## Materials and Methods

### Patients

This study was approved by the National Committee of Ethics and National Commission of Scientific Research of the Mexican Institute of Social Security (IMSS, 2008-785-066). Written informed consent was obtained from all participants, according to the Declaration of Helsinki. We obtained excisional biopsies from 15 hospitalized patients with diabetes and foot ulcer grade II according to Wagner's classification. Ulcers were debrided and patients were treated with systemic antibiotics for 7 days, cleaning the foot ulcer by strict washes every day until ulcer showed clinical improvement, then biopsy was taken and patients were followed to avoid complications. For healthy controls, normal skin fragments were taken from 15 clinically healthy individuals submitted to orthopedic surgery. None of these were IDDM or had any systemic or autoimmune disease; neither steroids nor any other hormone therapy was supplied. All patients were HIV negative. Clinical data is summarized in table 1.

**Table 1:** Clinical characteristics of the patients in each group.

Clinical characteristic	Healthy-non DM2 (15)	DM2 with foot ulceration (15)	Valor P
Age (years)	29±31	59±30	<0.003**
Gender (male/female)	12/3	9/6	–
BMI (kg/m^2^)	23.84±3.75	25.50±4.49	0.319
DM2 evolution (years)	NA	12.29±6.696	–
Glucose (mg/dl)	90.22±17.81	249.4±45.3	<0.001**

M, male; F, female.

NA  =  none applicable. All data are represented as mean ± SD except in the case of age, which is expressed as median ± IQR (interquartile range). p<0.05, ** p<0.01, *** p<0.001.

### Biopsies and primary epidermal keratinocytes culture

Excisional biopsies were obtained under local anaesthesia with 2% lidocaine from the border area of the ulcer and from similar anatomical site of non-diabetic subjects undergoing orthopaedic surgery. The obtained tissue corresponded to a rectangle of 10 mm of length for 5 mm wide, comprising the ulcer edge and surrounding skin. Each biopsy was divided in two halves; one was kept into RNAlater (Qiagen, Duesseldorf, Germany), whereas the other half was used to develop primary cell cultures, tissue was kept in medium DMEM-HAM-F12 for no longer than an hour. In order to discard bacterial infections, swabs for microbiology studies were obtained from all samples at the same time when biopsy was taken. The biopsies obtained from both groups were disaggregated mechanically with a scalp to obtain explants, which were seeded into cell culture flasks (Nunclon, Roskilde-Kamstrupyej, Denmark), with DMEM-HAM-F12 (Safc biosciences, Lenexa, Kansas, USA) to a proportion 1∶1 supplemented with 10% FBS (Gibco BRL, Carlsbad, CA, USA), 100 U.I/ml of penicillin and 100 μg/ml of streptomycin (Gibco, Carlsbad, CA) and incubated at 37°C with 5% CO_2_ atmosphere until adhesion. Once adhered, cells were supplemented with a keratinocyte growth kit (ATCC, Manassas, VA, USA) until cells reached 95% of confluence, which approximately took 30 days. Subsequently, the same supplemented cell culture media was used except for the antibiotics, mainly to avoid results variations during the antimicrobial activity assays.

### Phenotype identification, flow cytometry and stimulation

To confirm that primary epidermal cells were keratinocytes instead of fibroblasts, cell lysates were obtained and supplemented with proteases inhibitor (Sigma-Aldrich; St. Louis, USA). Equal amounts of total proteins were submitted to Western-blot analysis for cytokeratin-5 (KRT5) detection (Santa Cruz Biotechnology, Inc., Delaware, CA, USA), which is a protein specifically expressed in keratinocytes from the basal layer [24] using as endogen control β-actin (Sigma-Aldrich, St. Louis, USA).

The percentage of keratinocytes in cell cultures was determined by fluorescence-activated cell sorting (FACS) analysis using KRT5 antibody and isotype control. Briefly, cells were permeabilized with the fixation/permeabilization solution (BD-Pharmigen), washed, and incubated with a goat anti-KRT5 Ab (Santacruz Biotechnology, Santacruz, CA, USA), followed by a phycoerythrin (PE)-conjugated rabbit anti-goat IgG (Santacruz Biotechnology). Cells were analyzed in a FACSCanto II flow cytometer using the FACSDiva software (Becton Dickinson, San Jose, CA, USA). For each measurement a minimum of 10,000 cells was analyzed (Figure S3). Viability was checked by the Guava Viacount Assay (Millipore, Billerica, MA, USA) showing 95% of viability.

Once the cell phenotype and viability were confirmed, cells were treated with 50 µg/ml of L-isoleucine (Sigma-Aldrich, St. Louis, USA) for 18 hours. This concentration and time were established according to the result from a curve dose-response (Data not showed). Similarly, cells were stimulated with 10^−9^ M of active form vitamin D_3_ (1,25(OH)_2_D_3_)(Sigma-Aldrich, St. Louis, USA) or an equal amount of DMSO (Sigma-Aldrich, St. Louis, USA) (0.5% v/v, such as vehicle control) in 1% FBS for 18 hours [23]. After incubation, the obtained supernatants were supplemented with protease inhibitor cocktails, divided into aliquots and stored at −70°C until use.

### RNA isolation, reverse transcription and gene expression analysis determined by real time PCR

Total RNA from each primary culture was extracted with TRIzol (Gibco, Carlsbad, CA, USA) according to the manufacturer's instructions. Reverse mRNA transcription was performed using 1 µg total RNA, 2 µM Oligo (dT) 15 primer (Promega, Ontario, Canada), 10 units ribonuclease inhibitor (Invitrogen, Carslbard, CA) and 4 units Omniscript Reverse Transcriptase (Qiagen, Mexico). Real-time qPCR was performed using a LightCycler 2.0 thermocycler (Roche Applied Science Inc, USA), using specific hydrolysis probes and primers. Both were designed with universal probe library software (Roche Applied Science Inc., USA, see table 2). All data were analyzed using the expression of *hypoxanthine phosphoribosyl transferase (HPRT)* as a reference gene and internal endogen control. Relative quantification of gene expression was performed by the comparative quantification cycle (Cq) method, using the formula, 2^−ΔΔCT^ described previously by Livak and Schmittgen [25]. This method is based on the expression levels of a target gene versus one reference gene (*HPRT*) comparing between control group and target group. The comparative threshold cycle method was used to assess relative changes in mRNA levels between healthy individuals (control) reflected in fold changes. Thus healthy controls were normalized to one uniformly.

**Table 2:** Sequence of primers and hydrolysis probes used for real time-qPCR assays.

Gene name	Protein	Probe sequence	Right primer	Left primer
CAMP	LL-37	TCCAGGTC	GTCTGGGTCCCCATCCAT	TCGGATGCTAACCCTACG
DEFB4	HBD-2	TGTGGCTG	GAGGGAGCCCTTTTCTGAATC	GTCTCCCTGGAACAAAATGC
HPRT	HPRT	GCTGAGGA	CGAGCAAGACGTTCAGTCCT	TGACCTTGATTATTTTGCATACC
VDR	VDR	CATCACCA	AGGCTGCAAAGGCTTCTTC	ATGTCCACACAGCGTTTGAG

### Antimicrobial peptide quantification from cell-derived supernatants

The supernatants obtained from stimulated cells were collected as keratinocyte-conditioned medium (KCM) and filtrated using Amicon Ultra-4 centrifugal filter devices with a cut-off ≤10 kDa (Millipore, Billerica, MA, USA) according to the manufacturer's instructions and the flow-through was used for further analyses. Subsequently, we performed Bradford protein assay to measure total proteins in KCM and performed ELISA assay with equal amounts of total proteins. Briefly, ELISA plate (Maxisorb Immunoplate, Nunc and Wiesbaden, Germany) was covered with the capture antibody overnight (dil. 1∶500) (goat anti-LL-37 IgG, Santa Cruz Biotechnology, Inc., Delaware, CA, USA). After non-specific binding site were blocked, rabbit anti-LL-37 antibody (Abcam, Inc., Delaware, CA, USA) at a dilution of 1∶1000 was added and incubated overnight. Then washed and incubated with donkey anti-rabbit IgG biotin-labeled antibody (dil. 1∶500) (Santa Cruz Biotechnology, Inc., Delaware, CA, USA). The reaction was amplified adding avidin-biotin peroxidase (Biocare Medical, CA, USA). H_2_O_2_- 3,3′,5,5′- tetramethylbenzidine (Sigma-Aldrich, St. Louis, USA) was used as substrate. The concentration of LL-37 was calculated from the standard curve using synthesized LL-37 (Kindly donated by Dr. Robert Hancock, from British Columbia University, Vancouver Canada). For the specific case of HBD-2 it was used an ELISA kit from Peprotech (Rocky Hill, CT, USA) following manufacturer's recommendations.

### Antimicrobial assays

Microbial strain *E. coli* ATCC (67878) was purchased from the American Type Culture Collection. The multi-drug resistant *Pseudomonas aeruginosa* and *S. aureus* strains were isolated from diabetic foot ulcers and characterized by classical microbiological and biochemical methods.

Screening KCM to examine their antimicrobial activity was conducted using a modified radial diffusion assays. Standardized inoculum of each bacterium was spread on surfaces of Mueller-Hinton agar. 100 µl of KMC were put on agar surfaces to impregnate them. Amikacin (50 mg/ml) and synthetic peptides (solid-phase synthesized LL-37 (100 ng/ml) (kindly donated by Dr. Robert W. Hancock from British Columbia University) and recombinant HBD-2 (800 ng/ml) (peprotech, Mexico)) were used as positive controls and PBS1X was used as a negative control. Spread plates were kept at room temperature for 30 min to allow diffusion of each condition mentioned above prior to incubation at 37°C overnight. Following incubation, a clear area was formed (zone of inhibition) on the surface of the top agar representing inhibition of bacterial growth. The diameter of inhibition area was measured and reported in millimeters.

### In vitro wound closure assay

The in vitro wound closure assay was performed as described before [26]. Briefly, the human keratinocyte cell line HaCaT was seeded onto fibronectin-coated 24-well tissue culture plates in DMEM supplemented with 10% FBS until confluence. Then, the confluent monolayer of cells was denuded with a sterile 200-pipette tip to create a uniform cell-free zone in each well. The cultures were washed twice with PBS and re-coated with fibronectin (10 µg/ml) for 1 hour at 37°C. At this time point (t = 0 hours) wound margins were photo-documented. Then, cells were treated with LL-37 (100 ng/ml, diluted in cell culture media), HBD-2 (800 ng/ml, diluted in cell culture media), KCM and EGF (20 µg/ml) as positive control for up to 72 hours at 37°C and 5% CO_2_. Mitomycin C (10 µg/ml) was always included in the media to avoid cell proliferation. The repopulation of wounded areas was observed under an inverted microscope (Zeiss Axiovert 200M) and the same fields of the wound margin were photo-documented at different time points. Each image was measured using axiovision software. KCM was incubated with goat anti-LL-37 IgG (Santa Cruz Biotechnology, Inc., Delaware, CA, USA) and goat anti-HBD-2 (Santa Cruz Biotechnology, Inc., Delaware, CA, USA) for 2 hour at 4°C.

### Cell proliferation assay

Cell proliferation was measured using a BrdU cell proliferation enzyme-linked immunosorbent assay (ELISA) kit (Roche Molecular Biochemicals, Mannheim, Germany). The 5-bromo-2′-deoxyuridine (BrdU) assay was performed according to the manufacturer's protocol. Cells were incubated in media containing BrdU in the presence of KCM, EGF (20 µg/ml) as positive control and Mitomycin C (5 µg/ml) as negative control in a microtiter plate at a final volume of 200 µl/well for 24 h at 37°C. After removing labeling solution, cells were fixed in 200 µl FixDenat solution per well, incubated for 30 min at −20°C, and finally discarded. Nucleases were added for 30 min (37°C) to digest the BrdU-labeled DNA. Samples were then incubated for 30 min (37°C) with anti-BrdU-Peroxidase (POD) antibody. POD substrate was added and absorbance was measured after 15 min at 405 nm with a reference wavelength of 490 nm. The mean absorbance of the control cells represented 100% cell proliferation, and the mean absorbance of treated cells was related to control values to determine sensitivity. The BrdU assay was repeated in duplicate.

### Statistical analysis

Normality of all data was analyzed using a Kolmogorov-Smirnov normality test for each data set. Followed by non-parametric multiple comparison test Kruskal-Wallis to identify differences between groups. In the case of finding statistical significance (p<0.05) a Dunn's post hoc test was performed. For the analysis of clinical features, normality was verified using the same test after which a non-parametric of U Mann-Whitney test was used to identify differences between groups. Two-sided *p* values of <0.05 were considered statistically significant. Statistical analysis was performed using the GraphPad Prism Software (Graph Prism Software version 5.02, San Diego, CA).

## Results

### Individual's clinical data analysis

To discard the possibility that any clinical variant could interfere with the production of LL-37 and/or HBD-2 in both groups; main clinical parameters were compared between groups (Table 1). As expected, there were differences among the different groups regarding glucose levels *(p = 0.001)*. Age comparison between groups showed statistical differences *(p = 0.003)*. BMI was similar between the different groups showing a value of *p = 0.319*.

### Differential expression of DEFB4 and CAMP in primary epidermal cultures stimulated with 1,25(OH)_2_D_3_ and L-isoleucine from healthy donor biopsies

We performed primary cells cultures from DFU and healthy donor biopsies to determine that these cells were keratinocytes; a Western-blot assay and a FACS analysis for KRT5 were performed. Results showed that >95 of our cell cultures were keratinocytes (data not shown).

**Figure 1:**
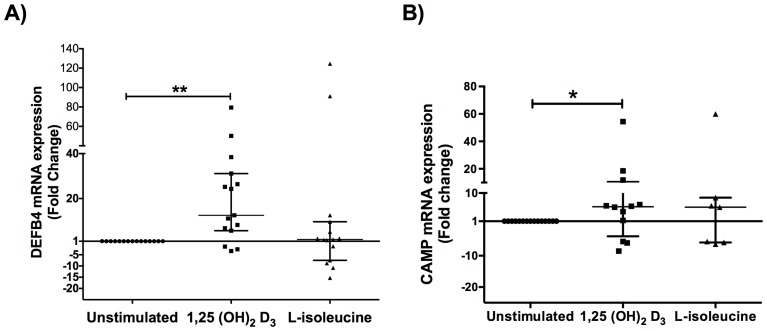
Antimicrobial peptides gene expression in healthy donors biopsies. Primary cells cultures from healthy individuals were performed and stimulated with L-isoleucine (50 µg/ml) and 1,25(OH)2D3 (10^−9^ M) for 18 h and DEFB4 (A) and CAMP (B) gene expression was measured by qPCR. mRNA levels of each AMP increased significantly after induction with 1,25(OH)2D3. Data is expressed as median ± interquartile range. Statistics were calculated by Kruskal-Wallis test. In each experimental group, n = 15. *p<0.05; ** p<0.01.

Real time qPCR analysis from healthy donors revealed that although L-isoleucine is able to induce DEFB4 gene expression in primary cultures, it showed no significant difference when compared with untreated group. Noteworthy, two primary cells cultures out of 15 had highest expression (more than 85 fold change) (**Figure 1A**). Similarly, when measured CAMP gene expression in the same samples we found that only 4 cell cultures responded to L-isoleucine treatment while the others samples showed no response (**Figure 1B**).

Interestingly, gene expression analysis from same cell cultures stimulated with 1,25(OH)_2_D_3_ showed that DEFB4 gene is increased in most samples. Indeed, 80% of cell cultures were responders, showing a ranging from 5.696 to 79.34-fold with *p* value of <0.01 compared with untreated cell cultures (**Figure 1A**). Besides, 60% of these cell cultures, showed an up-regulation regarding CAMP gene expression ranging from 1.266 to 54.38-fold when compared with untreated cell cultures *p*<0.05 (**Figure 1B**). In summary, 1,25(OH)_2_D_3_ is able to modulate DEFB4 and CAMP gene expression in primary cultures from healthy donors.

### Differential expression of DEFB4 and CAMP in primary epidermal cultures from DFU biopsies, stimulated with L-isoleucine and 1,25(OH)_2_D_3_

We recently showed that patients with DFU have lower levels DEFB4 in comparison with healthy skin. Furthermore, most of the DFU patients showed a lack of CAMP gene expression [16], suggesting that low levels of these peptides contribute to poor wound healing in DFUs. In this study, we examined whether L-isoleucine and 1,25 (OH)_2_D_3_ were capable to restore this deficiency.

**Figure 2:**
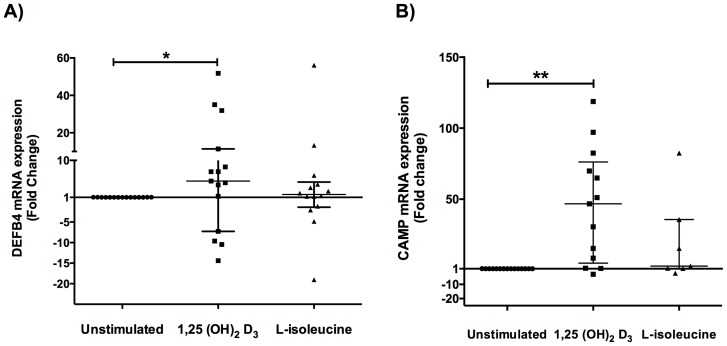
Antimicrobial peptides gene expression in cell cultures from DFU biopsies. Primary cells cultures from diabetic foot ulcers biopsies were performed and stimulated with L-isoleucine (50 µg/ml) and 1,25(OH)2D3 (10^−9^ M) for 18 h and DEFB4 (A) and CAMP (B) gene expression was measured by qPCR. Data are expressed as median ± interquartile range. Statistics were calculated by Kruskal-Wallis test. In each experimental group, n = 15. *p<0.05; ** p<0.01.

Real time qPCR gene expression analysis showed that L-isoleucine was able to induce DEFB4 gene expression varying from 1.223 to 56.10-fold versus no treated cells. Nevertheless, 73.3% of same primary cultures responded to 1,25(OH)_2_D_3_ stimulation, increasing gene expression of this peptide within ranges from 1.2 to 51.8-fold with *p* value of <0.05 (**Figure 2A**). In regards with CRAMP, when primary cell cultures were stimulated with L-isoleucine, only six cultures responded to the treatment increasing modestly the gene expression whereas 80% of these cultures responded to 1,25(OH)_2_D_3_ increasing the gene expression form 1.20 to 118.80-fold when compared with untreated cell cultures (**Figure 2B**). These results demonstrate that the gene expression of each AMPs is restore under the treatment with 1,25(OH)_2_D_3_ in primary cultures of skin biopsies from DFU.

### 1,25(OH)_2_D_3_ induces changes in the secretion of HBD-2 and LL-37 in supernatants from primary cells cultures from DFU

**Figure 3:**
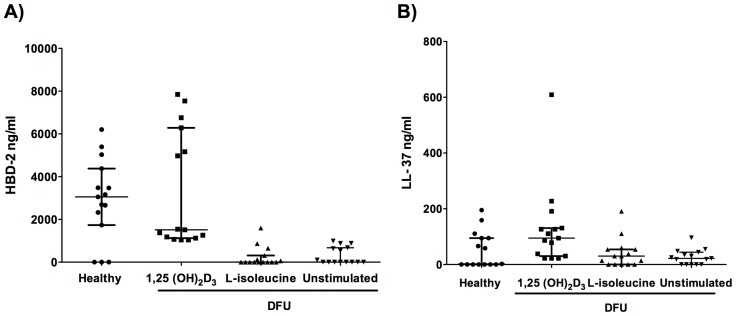
Production of HBD-2 and LL-37 in primary cell cultures from healthy donors and DFU patient stimulated with 1,25(OH)2D3 and L-isoleucine. HBD-2 (A) and LL-37 (B) production was measured by ELISA assay from supernant concentrates. Data is expressed as median ± interquartile range. Statistics were calculated by Kruskal-Wallis test. In each experimental group, n = 15.

Given that not all transcribed mRNA is traduced into AMPs, we determined the production LL-37 and HBD-2 in KCM from primary cells cultures by Enzyme-linked Immunosorbent Assay (ELISA). The results showed that 1,25(OH)_2_D_3_ induced HBD-2 secretion in 5 out of 15 cell cultures from DFUs reaching levels up to 8000 ng/ml even above of the levels seen for the cell culture stimulated from healthy donors. Whereas, L-isoleucine treatment induced a mild HBD-2 production only in 3 out of 15 cell cultures from DFU (**Figure 3a**).

The induction with L-isoleucine in cell cultures from DFU resulted into a mild production of LL-37, which reached the basal production seen in healthy controls. Following stimulation with 1,25(OH)_2_D_3_, 60% of these cultures achieve amounts above 100 ng/ml of LL-37, some of them reaching similar values to those detected for healthy controls (**Figure 3B**). In summary, our results suggest that 1,25(OH)_2_D_3_ restores the production of AMPs in primary cell from DFU which could be involved in wounding activities.

### Gene expression of VDR is not altered in diabetic foot ulcers biopsies

**Figure 4:**
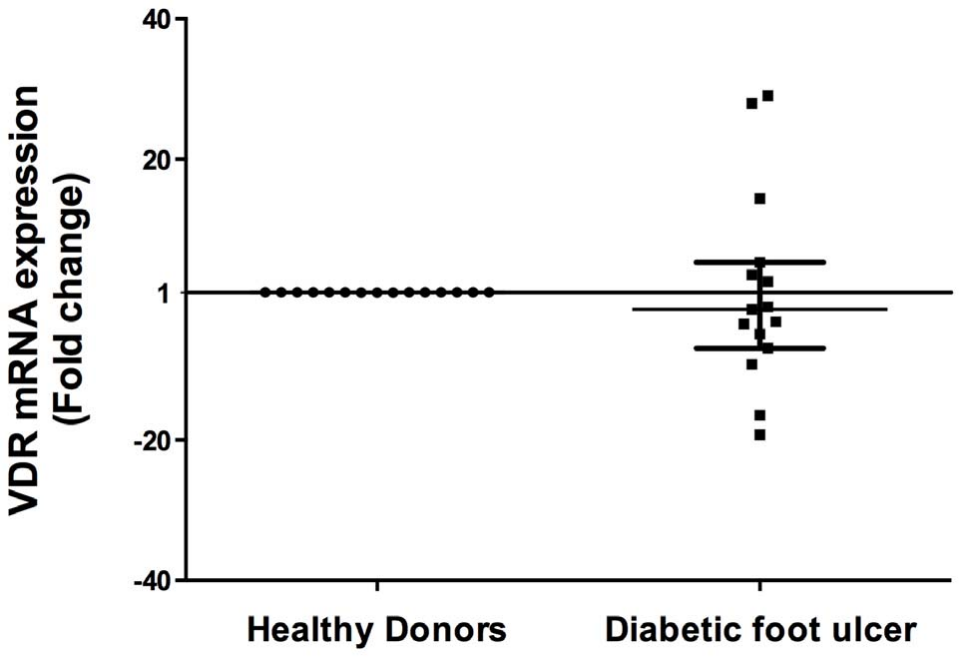
Vitamin D receptor gene expression in diabetic foot ulcers biopsies. Real time PCR was performed from DFU and healthy donors biopsies. Results show mRNA levels of VDR compared with healthy individuals are not statistically significant. Data is expressed as median ± interquartile range. Statistics were calculated by Mann Whitney test. In each experimental group, n = 15.

It has been reported that 1,25(OH)_2_D_3_ signals through the vitamin D receptor, a ligand-stimulated transcription factor that recognizes specific DNA sequences called vitamin D response elements which are located in the promoter regions from DEFB4 and CAMP [23]. Since not all cell cultures from DFU responded properly to 1,25(OH)_2_D_3_ stimulation, we wondered whether there was a failure in the VDR expression. Our results showed that there are not significant differences between biopsies from DFU and healthy skin regarding VDR gene expression (*p* = 0.34) (**Figure 4A**).

### Keratinocyte-conditioned medium mediate antimicrobial effect over *E. coli*

**Figure 5:**
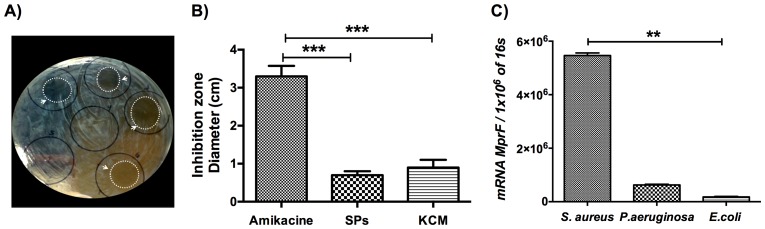
KCM antimicrobial activity in *E. coli*. KCMs from DFUs that showed higher LL-37 and HBD-2 concentrations were tested for antimicrobial activity against *S. aureus, P. aeruginosa* and *E. coli* in a modified radial diffusion assay. Eight KCMs from DFUs cell cultures showed clear inhibition areas (arrow heads) only against *E. coli* (A and B) comparable with the solid-phase synthesized LL-37 (SP). *mprf* gene expression in the different clinical isolates (C). Each bar represents the mean ± SD of five independent experiments.

We collected eight (KCM) from vitamin D- treated DFUs cell cultures, which responded favorably to the treatment reflected by the LL-37 production and we aimed to determine the antimicrobial activity of these KCMs against most common strains. Thus, we isolated and characterized *P. aeruginosa* and *S. aureus* strains from ulcerative exudates. Screening KCM for their antibacterial activity was conducted using radial diffusion assays. The results showed that KCM did not result in larger zones of inhibition against clinical isolates neither inhibition areas were observed with synthetic peptides (Figure S1), suggesting that both strains have some biological component that confers its resistance. Therefore, we used *E. coli* as reference strain to verify the above observation. The results demonstrated that these supernatants showed a clear inhibition area against *E. coli* (1.033±0.1614 cm diameter) (**Figure 5A–B**). Since not all KCM showed antimicrobial activity against clinical isolates, we wondered whether clinical isolates had some resistance gene for AMPs antimicrobial activity. Interestingly, we found multiple peptide resistance factor (*mprf*) gene in both clinical isolates, *S. aureus* strain has a higher copy number than *P. aeruginosa* (**Figure 5C**).

### Keratinocyte-conditioned medium promote wound closure and increases cell proliferation

In addition to its antimicrobial action, other activities have been associated to LL-37 and HBD-2 such as keratinocyte migration during wound healing processes [27], we hypothesized that these 1,25(OH)_2_D_3_-inducible peptides could also stimulate HaCaT migration promoting wound closure. We tested the supernatants from DFUs stimulated with 1,25 (OH)_2_D_3_ with concentrations above 100 ng/ml of LL-37 and above 5 µg of HBD-2. The KCM were tested in an in vitro wound-healing assay.

**Figure 6:**
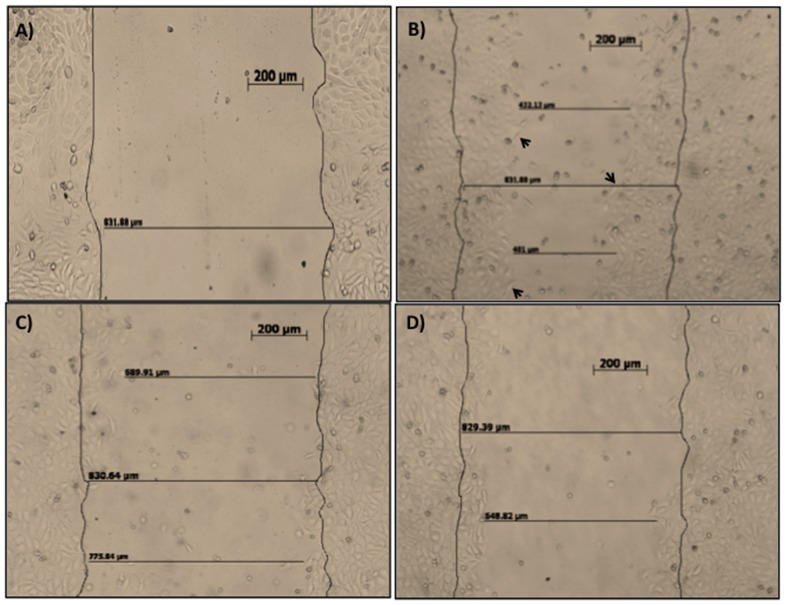
Keratinocyte-conditioned medium mediate keratinocyte migration (in vitro wound closure assay). HaCaT cells were grown to confluence on 24-well tissue culture plates. Then each well was denuded by half from cells and re-coated with fibronectin. The cells were incubated with KCM from DFUs, 0 h. (6A); KCM, 48 h. (6B); KCM with goat polyclonal anti-HBD-2, 48 h (6C); KCM with goat polyclonal anti-LL-37 (6D). Migrations were photographed and monitored for up to 48 h. Data shown are representative of three independent experiments. Bar = 200 µm.

Results showed that KCM from DFUs induced keratinocyte migration mainly at 48 hours post-stimulation (**Figure 6A–B**), worthwhile to mention that migrating cells had an evident invasive phenotype, which is seen during in vivo wound healing (**Figure 6B**, arrow heads). To demonstrate the role of HBD-2 and LL-37 present in the KCM to induce migration, we used blocking antibodies for HBD-2 (**Figure 6C**) or for LL-37 (**Figure 6D**). Results showed that blocking antibodies abrogated cell migration. Control experiments using EGF, HBD-2 and LL-37 alone, show the expected proliferation (Figure S2).

**Figure 7:**
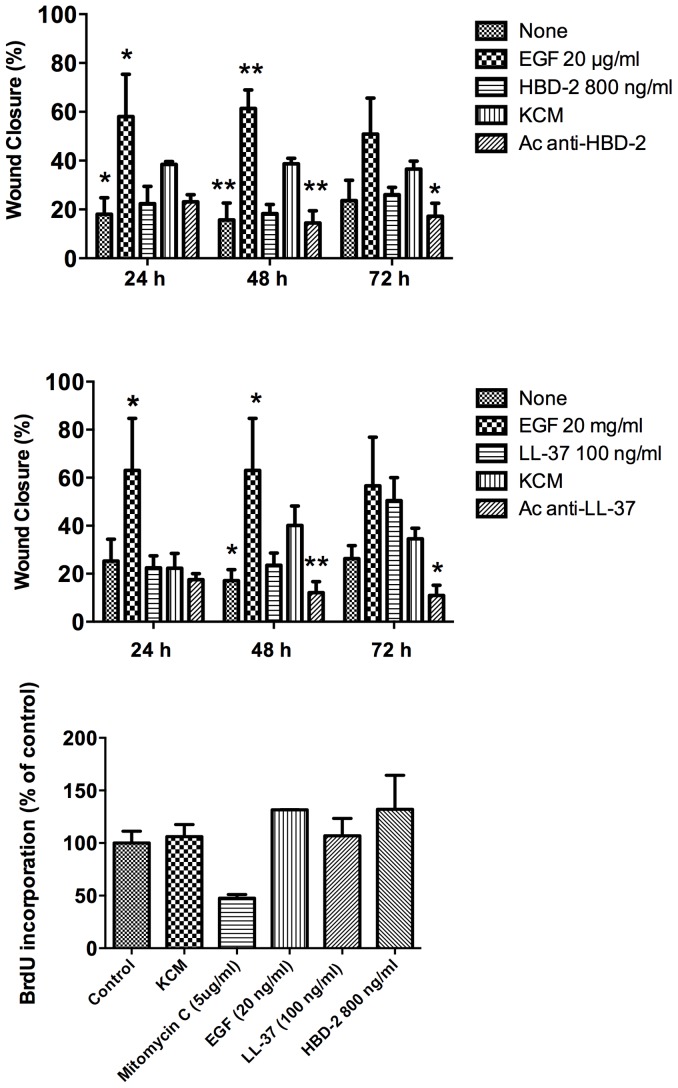
Keratinocyte-conditioned medium mediate proliferation and keratinocyte migration. Vitamin D- treated KCM from DFU were tested for cell migration induction. HaCaT cells were grown to confluence on 24-well tissue culture plates. Then each well was denuded by half from cells and re-coated with fibronectin, then we measured wound closure at 24, 48 and 72 hours post-incubation using as controls Epithelial Growth Factor (EGF), LL-37 or HBD-2, and the KCM with blocking antibodies either for HBD-2 (7A) or for LL-37 (7B). BrdU incorporation was used as an index of cellular proliferation (7C). Data are expressed as a percentage of the BrdU-positive cells (BrdU incorporation). Each value represents the median ± interquartile range. Values are the mean of 3 independent experiments. Asterisks show the treated groups with statistical difference when compared with KCM for each time point. * p<0.05, ** p<0.01.

Subsequently, we measured the percentage of wound closure at 24, 48 and 72 hours post-incubation. Results showed that KCMs increase wound closure reaching 40%, whereas none-treated cultures reached 18–22%. Positive control (EGF) reached percentages above 55%. To evaluate whether this activity is AMPs-dependent, we blocked LL-37 and HBD-2 with specific antibodies. Results showed that the use of blocking antibodies decreases wound closure for both LL-37 and HBD-2, albeit is more evident for the case of LL-37 (**Figure 7A and 7B**).

On the other hand, another important stage during wound healing is the keratinocyte proliferation, which is evident mainly at the first 24 hours post-injury. We wondered whether the KCM could induce keratinocyte proliferation, thus cell proliferation was evaluated through BrdU incorporation. Our results showed that KCM induce proliferation alike the positive control (EGF) and the synthetic peptides though is not significative suggest that KCM could maintain viable cells and promote re-epithelization of wound through keratinocyte migration.

## Discussion

Most foot problems that people with diabetes face, arise from two serious complications of this disease: poor wound healing and reduced blood flow which can allow a small blister to progress to a serious infection in a matter of days which finally leads to amputations in most of the cases [28]. In the present study we aimed to determine whether LL-37 and/or HBD-2 could be induced with 1,25(OH)_2_D_3_ or with L-isoleucine due both peptides have antimicrobial, angiogenic and wound healing properties. We took biopsies from patients with DFU grade II according Wagner's classification and healthy donors, our clinical analysis showed that only the age and glucose levels were statistically significant between groups. Both age and glucose levels have not been clearly associated with AMPs decreased production at the skin level. However, studies by our group showed that diabetic patients have lower levels of LL-37 and HBD-2 either in peripheral blood mononuclear cells [15] or skin [16] when compared with healthy donors. This data suggests that increasing glucose levels may be related with a low production of AMPs. Regarding age, recent studies have shown that there is not significant reduction of AMPs during ageing [29].

Wound healing in DFU is a complex process involving multiple cell features, such as migration, proliferation and differentiation of the dermal fibroblast and epidermal keratinocytes [30]. All of these steps involve a well-orchestrated regulation of multiple signaling pathways that control the expression of many genes endowed with diverse crucial functions such as AMPs [7]. Interestingly, previous studies done by our group, demonstrated that biopsies from DFU had low or none cathelicidin expression [16]. This absence of LL-37 may contribute directly with the delayed or incapacity of DFU for wound healing. This information motivated us to enquire whether AMPs could be induced with the active form of vitamin D_3_ known as 1,25-dihydroxycholecalciferol 1,25(OH)_2_D_3_ or L-isoleucine, since it has been reported elsewhere that these molecules induced high levels of these peptides in monocytes [31], skin [32], lung [17], sebocytes [21] and kidney [19].

In the first instance, we performed primary cultures from healthy donors skin biopsies to assess the proper functioning of the induction system. Our results showed that primary cultures obtained and then stimulated with L-isoleucine are capable to induce cathelicidin and HBD-2 gene expression, while 1,25(OH)_2_D_3_ increased the gene expression of DEFB4 and CAMP in comparison with untreated cultures; suggesting that 1,25(OH)_2_D_3_ is an efficient inductor of AMPs in keratinocytes from healthy donors.

Thus the next question was whether 1,25(OH)_2_D_3_ was able to induce AMPs in DFUs, to achieve this, we performed primary cultures from DFUs and stimulated them with the inductors above mentioned. The result showed that L-isoleucine was able to induce DEFB4 expression in 50% of the treated cultures; however only few of the treated cultures showed CAMP gene expression. Nonetheless, results showed that 1,25(OH)_2_D_3_ was able to induce the expression of both CAMP and DEFB4 in 13 out of 15 cell cultures ranging from 2 to 125 fold changes. Our ELISA studies correlates with the gene expression analysis, showing that patients that had higher expression correlated with higher concentrations of each peptide in supernatant.

Intriguingly, previous studies by our group showed that most DFU patients showed not CAMP expression in biopsies from the ulcer [16], thus, these results may suggest that production of cathelicidin can be retrieved for DFU patients using 1,25(OH)_2_D_3_ treatment.

Since we observed that some cell cultures had low or not response at all to 1,25(OH)_2_D_3_, we wonder whether VDR which is a nuclear receptor that renders responsiveness to vitamin D_3_ and is required for an accurate AMP expression was expressed properly in these patients. Our results indicate that there are no statistically significant differences between healthy donors and biopsies from DFU patients regarding VDR expression. This suggested that the lack or low responsiveness between individuals could be related to SNPs in VDR though further studies need to be done.

On the other hand, when supernatants from these DFU treated-cultures were tested for antimicrobial activity against clinical isolates, the results showed none antimicrobial inhibition, the same effect was observed when used the synthetic peptide with biological activity. Interestingly, both clinical isolated strains had the gene MprF, which mediates resistance to AMPs through the modification of the membrane lipid phosphatidylglycerol (PG) by enzymatic transfer of a L-lysine residue, leading to lysyl-PG converting the net charge of PG from −1 to +1 [33]. In the particular case of *P. aeruginosa* MprF1 is specific for Ala-tRNA (Ala) that provides a mechanism by which the cell can fine-tune the charge of the inner membrane by using the neutral amino acid alanine, potentially providing resistance to a broader range of AMPs than offered by lysine modification alone [34]–[36]. Although this has not been reported in the DFU pathology we assume that is an important pathogenic mechanism of *P. aeruginosa* in this particular pathology, however, further studies need to be done. Therefore, the antimicrobial activity of 1,25(OH)_2_D_3_-inducible peptides in this assay were blocked in the presence of this gene. However, when same supernatants were used to test antimicrobial activity against *E. coli*, they showed a mild antimicrobial effect, suggesting that presence of MprF decrease antimicrobial activity of KCM. Nevertheless, in separate experiments, same supernatants clearly induced a migratory phenotype in HaCaT cells such as an epithelial-mesenchymal transition (EMT) which is characterized by the acquisition of mesenchymal properties linked with an invasive phenotype. This phenomenon was described previously, where participation of the Snail family have been implicated in the cellular acquisition of migratory properties following down regulation of expression of the adhesion protein E-cadherin and the loss of E-cadherin in EMT is frequently due to the transcriptional family Snail [37]. Although we do not determine the expression of Slug, a member of the snail family of transcription factors that was reported to be upregulated in keratinocytes during wound healing [38]. Carretero et al., described that LL-37 synthetic peptide was capable to induce Snail and Slug mRNA in HaCaT cells treated [26].

We suggest that the reason of why we found migration activity and not a strong antimicrobial activity is because for antimicrobial activity the amount of AMPs must be higher than for wounding activity according to previous studies [10], [26].

Cathelicidin promotes wound healing by increasing the re-epithelialization rate and granulation tissue formation, since induces keratinocyte migration through FPRL-1 signaling or transactivation of EGFR [39], therefore, due to these pro-wounding functions, cathelicidin is apparently essential for a proper wound healing process. Interestingly, our study demonstrated that 60% of the cell cultures secreted 100 ng/ml of LL-37, which is sufficient to promote migration of keratinocytes during wound healing process [26]. In this study, we observed that the primary cultures that produced more efficiently HBD-2 and LL-37 are derived from patients who take a good glycemic control and few years of NIDDM evolution, therefore this data suggest that probably FPRL-1 migration-related receptor could be glycated, however this must be further elucidated.

Besides that LL-37 and HBD-2 have wounding activities by themselves, they may induce other components needed for this wound healing [40]. In the present study it was demonstrated that KCM increased cellular proliferation modestly, however had no significant difference when compared with EGF or the synthetic peptide, this data suggest that the modest proliferation could be maintaining viable cells and promote re-epithelization of wound through keratinocyte migration. Worthwhile to notice that EGF treated keratinocytes seem to stagnate in migration after day 1 and did not reach 100% within 72 hours, this could be explained because the number of EGF receptors in cells surface treated constantly with EGF is downregulated as negative feedback. Actually, stimulation with 100 ng/ml EGF caused aggregation of the EGF associated with the plasma membrane, followed by receptor internalization at longer stimulation times [41]. Indeed we used 20 μg/ml, which definitely could lead to the receptor saturation promoting internalization and unresponsiveness.

In summary, 1,25 (OH)_2_D_3_ is able to induce gene expression and production of HBD-2 and LL-37 in primary keratinocytes culture from DFU and that KCM from stimulated cells induce keratinocyte migration and proliferation, suggesting their possible therapeutic use in the treatment of diabetic foot ulcers as low-cost alternative.

## Supporting Information

Figure S1 **KCM antimicrobial activity in clinical Isolates.** KCM from DFUs which showed higher LL-37 and HBD-2 concentration were tested for antimicrobial activity in *S. aureus* (A) and *in P. aeruginosa* (B).
(TIF)

Figure S2 **Control experiments.** Control experiments using EGF, HBD-2 and LL-37 alone show the expected proliferation.
(TIF)

Figure S3 **Phenotype evaluation.** To confirm that primary epidermal cells were keratinocytes instead of fibroblasts we checked the cell culture morphology, which corresponded to basal keratinocytes (Panel A). To confirm the phenotype, we performed an immunocytochemistry assay to detect cytokeratin-5 (KRT) which is a protein specifically expressed in keratinocytes from the basal layer (Panel B). Besides we obtained cell lysates from cultures, equal amounts of total proteins were submitted to Western-blot analysis using as endogen control β-actin, results showed that all cell cultures were positive to KRT5 (Panel C). Once we knew that all cultures expressed KRT5, we determined the percentage of cells positive for KRT5 by flow cytometry showing percentages >95% (Panel D).
(TIF)
