# Supplementary figures and images for: 1,25-Dihydroxyvitamin D3 Induces LL-37 and HBD-2 Production in Keratinocytes from Diabetic Foot Ulcers Promoting Wound Healing: An In Vitro Model
Source: PLoS One. 2014 Oct 22;9(10):e111355. doi: 10.1371/journal.pone.0111355 (PMC4206472; doi:10.1371/journal.pone.0111355)

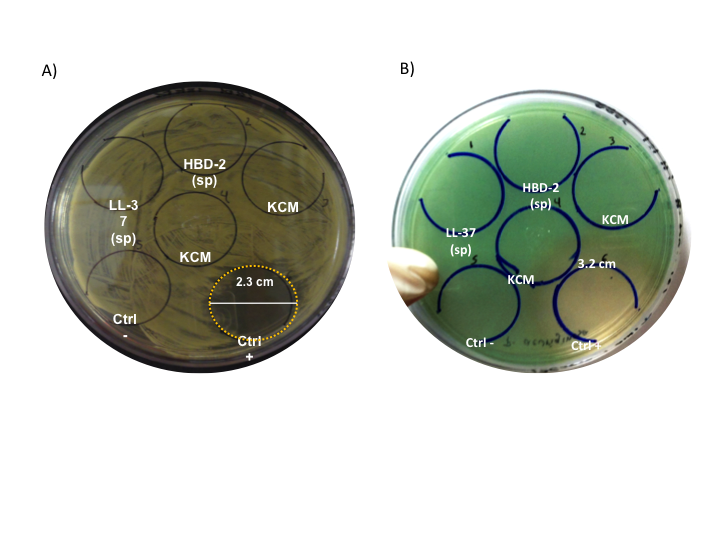

Supplement: Figure S1 — KCM antimicrobial activity in clinical Isolates. KCM from DFUs which showed higher LL-37 and HBD-2 concentration were tested for antimicrobial activity in S. aureus (A) and in P. aeruginosa (B). (TIF) [file pone.0111355.s001.tif]

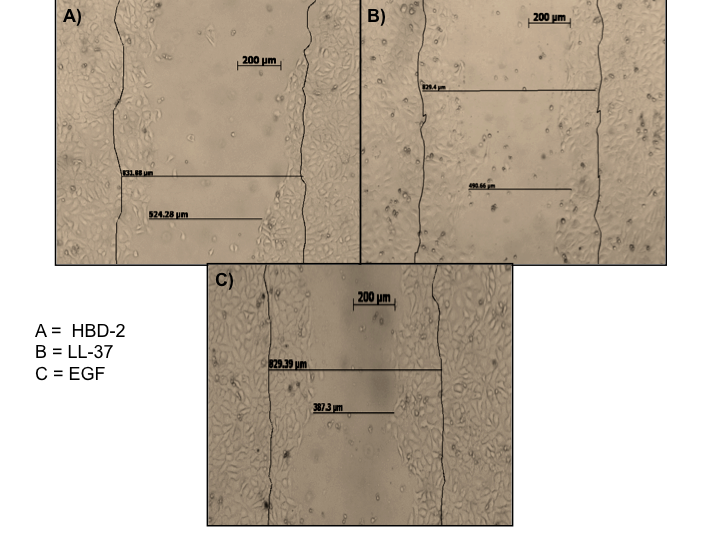

Supplement: Figure S2 — Control experiments. Control experiments using EGF, HBD-2 and LL-37 alone show the expected proliferation. (TIF) [file pone.0111355.s002.tif]

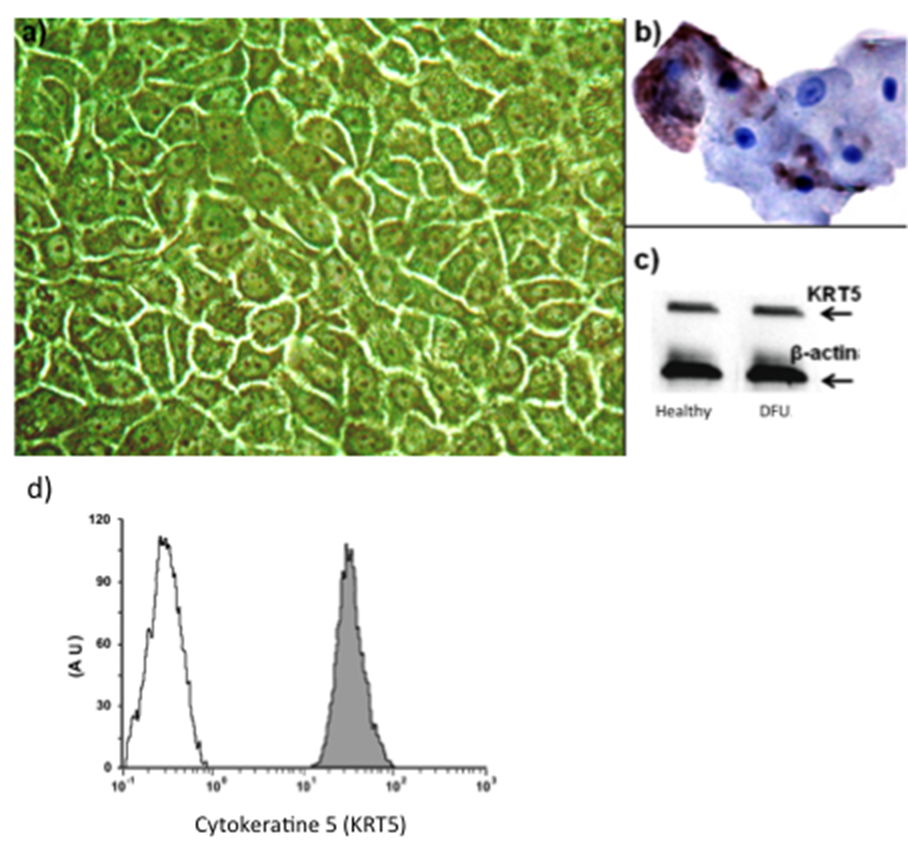

Supplement: Figure S3 — Phenotype evaluation. To confirm that primary epidermal cells were keratinocytes instead of fibroblasts we checked the cell culture morphology, which corresponded to basal keratinocytes (Panel A). To confirm the phenotype, we performed an immunocytochemistry assay to detect cytokeratin-5 (KRT) which is a protein specifically expressed in keratinocytes from the basal layer (Panel B). Besides we obtained cell lysates from cultures, equal amounts of total proteins were submitted to Western-blot analysis using as endogen control β-actin, results showed that all cell cultures were positive to KRT5 (Panel C). Once we knew that all cultures expressed KRT5, we determined the percentage of cells positive for KRT5 by flow cytometry showing percentages >95% (Panel D). (TIF) [file pone.0111355.s003.tif]
